# Effect of Body Configuration on Perturbation Resistance Across Arboreal Lizard Species

**DOI:** 10.1002/jez.70041

**Published:** 2025-10-15

**Authors:** Victor David Munteanu, Trevor Brewington, Savannah Swisher, Amanda Kellerhals, Richard W. Blob

**Affiliations:** ^1^ Department of Biological Sciences Clemson University Clemson South Carolina USA

**Keywords:** biomechanics, kinematics, stability

## Abstract

Animals that inhabit high‐risk habitats often exhibit morphological and behavioral adaptations to contend with environmental challenges. In arboreal (tree‐based) habitats, such adaptations can include corrective behaviors, such as modification of body‐limb angles, to avoid dislodgement by perturbations. This study evaluated shifts in limb and tail movements by three arboreal lizard species with different body configurations (variations of limb posture, body height, and tail prehensility) as they experienced simulations of unexpected arboreal perturbations. Animals were placed on a custom‐built, laterally sliding perch apparatus, with trials filmed using high‐speed video. Effects of different body configuration on restabilization performance were evaluated by comparing center of mass (CoM) displacement, limb angles, and tail behavior that occurred during the recovery from a sudden stoppage of perch movement. Results indicated that both body configuration and tail behavior influenced CoM displacement more than other kinematic factors. Across the three configurations that we compared, the sprawling, prehensile‐tailed body configuration showed significantly larger CoM displacement compared to the upright, prehensile‐tailed and the sprawling, non‐prehensile‐tailed configurations, especially when utilizing dynamic tail rotation as a stabilization behavior. These data indicate that a wide range of kinematic behaviors can be employed by arboreal lizards to ensure stability when subjected to potential dislodgement, but specific approaches, may contribute to superior performance for species with particular body designs, as seen with the use of dynamic tail rotation used by sprawling, prehensile‐tailed species.

## Summary

This study evaluated the restabilizing behaviors of arboreal lizards when subjected to a simulated environmental perturbation. Results found that body configuration and tail behavior have strong influence on restabilization performance.

## 1 Introduction

The morphological diversity of organisms is commonly viewed as reflecting the range of distinct functional demands that they encounter across diverse environments (Wainwright 2005; Olsen 2017; Williams 2019; Sommer and Wehner 2012; Moon et al. 2019). The physical features of a habitat can impose selective pressures, under which organisms with more advantageous traits might have greater success in acquiring resources and reproducing, impacting the success of lineages through evolutionary time (Blob et al. 2008; Bodensteiner et al. 2021; Huey et al. 2009). This framework can contribute to a lineage specializing for the demands of a particular environment, or help to determine which lineages are successful during invasions of novel habitats (Wainwright 2005; Young and Blob 2015).

Beyond the pressures of any predominating environmental conditions, in many natural habitats animals may encounter conditions or need to traverse substrates that are unstable, threatening equilibrium and steady locomotion. Behaviors that enable locomotion despite such threats to stability can be crucial for survival, preventing injury or displacement from intended paths of travel. Such behaviors have been documented in aquatic, terrestrial, and aerial contexts (Boerma et al. 2019; Clark and Higham 2011; Combes et al. 2023; Daley and Biewener 2006; Fish 2002; Hedrick and Daniel 2006; Rivera et al. 2011). For instance, Atlantic brief squid (*Lolliguncula brevis*) were found to use their mantle fins in ways that can optimize stability when traversing aquatic habitats (Bartol et al. 2022). On land, sprinting guinea fowl (*Numida meleagris*) that experience a sudden descent in substrate height can modify locomotor kinematics to maintain equilibrium and avoid falling (Daley and Biewener 2006). Among flying animals, bats that experience a jet of air mid‐flight are able to use their wings as an inertial appendage to restabilize and return to steady locomotion (Boerma et al. 2019). This diversity of examples across habitats and species illustrates the ubiquity of independently derived stabilizing behaviors employed by moving animals.

Arboreal, or tree‐based habitats, can be complex environments with a multitude of surface geometries, textures, angles, and discontinuities all presenting novel locomotor tasks that potential inhabitants must contend with to successfully move through and persist in such conditions (Dufour et al. 2019; Graham and Socha 2020; Shattuck and Williams 2010; Young et al. 2015). Such novel physical demands can have profound consequences for the evolution of morphology, performance, and behavior in lineages. There are several instances of distinctive morphological traits in arboreal animals that appear related to specific ecological demands of such habitats. For example, longer tails (Mincer and Russo 2020), as well as static and dynamic tail usage (in both prehensile and non‐prehensile contexts) may help animals resist falling in unstable arboreal environments (Lemelin 1995; Böhmer et al. 2019; Hager and Hoekstra 2021; Young et al. 2021). In addition to tail use, animals also often exhibit specializations in their hand and foot morphologies which assist in gripping variable surface geometries (Böhmer et al. 2019; DiPaolo and Kolbe 2023). Within the vertebrate lineages that have invaded arboreal habitats, behavioral and morphological traits are often employed in conjunction to avoid displacement from trees, which may relate to the potentially high risks of injury or death associated with falling (Nakai 2003; Jusufi et al. 2011; Jurmain 1997). With the potential severity of such risks, strong selection seems likely to have operated on morphological and behavioral traits that would reduce instability and the risks it might impose. But within this context, arboreal animals still exhibit a diverse range of body configurations and behaviors (Jusufi et al. 2008; Nyakatura and Fischer 2010; Young et al. 2015). As a result, comparisons of stabilization performance in arboreal habitats provide an opportunity to understand what specializations may develop under strong environmental selective pressures, and how animals contend with instability.

Within lizards, multiple lineages have independently invaded arboreal habitats, including species that exhibit morphologies distinctly specialized for arboreality as well as species that remain similar in appearance to those found in related, more terrestrial, taxa (Irschick and Losos 1999; Autumn et al. 2006; Herrel et al. 2013). Given the range of body plans and independent origins of arboreality exhibited by lizards, comparisons of their performance in association with the risks and selective pressures of arboreal habitats provide an opportunity to gain novel insights into how diverse features contribute to the successful use of such environments.

**Figure 1:**
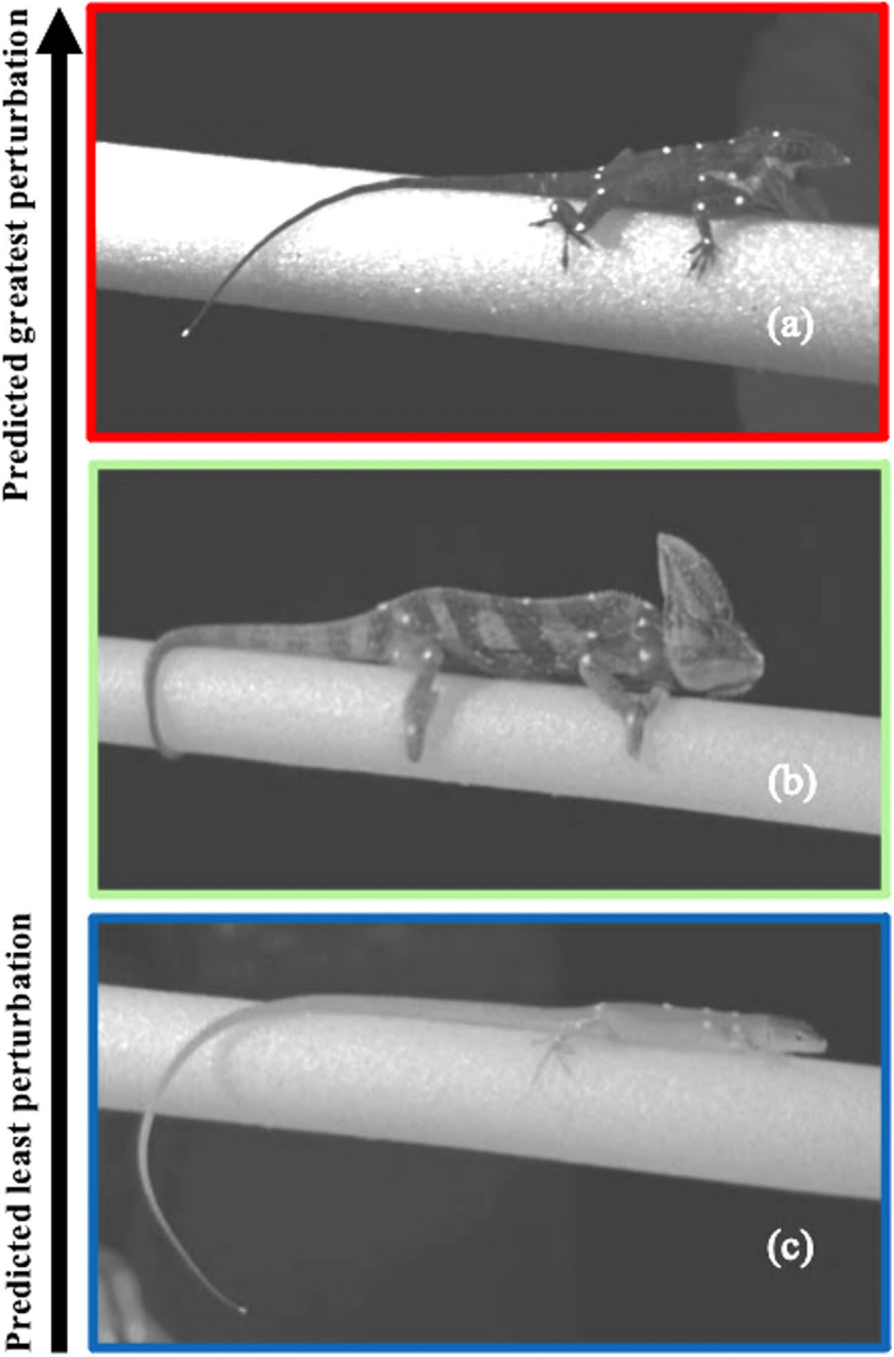
Representative species (a) *Anolis equestris* (sprawling non‐prehensile‐tailed body configuration); (b) *Chamaeleo calyptratus* (upright prehensile‐tailed body configuration); and (c) *Gastropholis prasina* (sprawling prehensile‐tailed body configuration). Animals ordered in predicted magnitude of perturbation across stabilization metrics, from low (bottom panel) to high (top panel). Images have been digitally brightened.

In this study, we investigated arboreal lizards' ability to contend with simulated arboreal perturbation. We used high‐speed video to compare how arboreal lizards with different body designs contend with perch perturbations that simulate environmental disturbances that animals may experience in nature. We used three species (Figure 1) as models to represent three distinct body configurations, respectively: (1) *Anolis equestris*: non‐prehensile‐tailed with sprawling limb posture and a low center of mass (CoM); (2) *Gastropholis prasina*: prehensile‐tailed with sprawling limb posture and a low CoM; and (3) *Chamaeleo calyptratus*: prehensile‐tailed with parasagittal limbs and a high CoM. These species represent different lizard clades (iguanids, lacertids and chameleonids, respectively), providing a broad phylogenetic scope for our comparisons. These three lineages appear to show independent origins of arboreal habits based on their phylogenetic associations (Simões and Pyron 2021). We predict that *Anolis*, the lizard species with a low CoM and non‐prehensile‐tailed configuration, will show the greatest disruption from their perch during a perturbation, followed by *Chamaeleo* with a high CoM and prehensile tail, and then by *Gastropholis* with a low CoM and prehensile tail. Our basis for this prediction is that both CoM height and the presence of accessory structures could improve resistance to dislodgement during a perturbation. Maintaining a CoM closer to the substrate is commonly observed in above‐branch stance and locomotion among other arboreal animals, potentially helping to maintain equilibrium (Cartmill 1985; Chadwell and Young 2015; Van Damme 1997). In addition, accessory gripping of branches by prehensile tails could further help maintain balance (Hager and Hoekstra 2021). We predict that these characteristics will work in concert to amplify stabilization in arboreal habitats.

## 2 Materials andMethods

### 2.1 Study Animals and Husbandry

*Anolis equestris* (*n* = 3), *Gastropholis prasina* (*n* = 3), and *Chamaeleo calyptratus* (*n* = 3), lizards were procured from commercial breeders. Only male animals were used to eliminate variation across individuals potentially related to sexual dimorphism. These species are comparable to each other in body mass and snout‐vent length, and body sizes were closely matched across individuals of the same species (*Anolis* mass = 15 ± 6 g; *Gastropholis* = 28 ± 4 g; *Chamaeleo* = 102 ± 58 g). Results were normalized for body size differences as appropriate (see below). Animals were housed in species‐specific enclosures in our home lab facility in Clemson, SC, USA (45 cm L × 90 cm W × 90 cm H for *C. calyptratus*, 45 cm L × 45 cm W × 90 cm H for the other two species). Each enclosure was fitted with climbing surfaces, basking areas, and shelters to promote activity and enrichment. Ambient temperatures were kept 23°C–29°C with basking areas reaching 33°C. Ambient, UV, and basking lighting was maintained on a 12:12 light:dark cycle. Animals were supplied water three times daily via a misting system and were fed three times per week with vitamin‐dusted insects.

### 2.2 Test Apparatus Design and Construction

**Figure 2:**
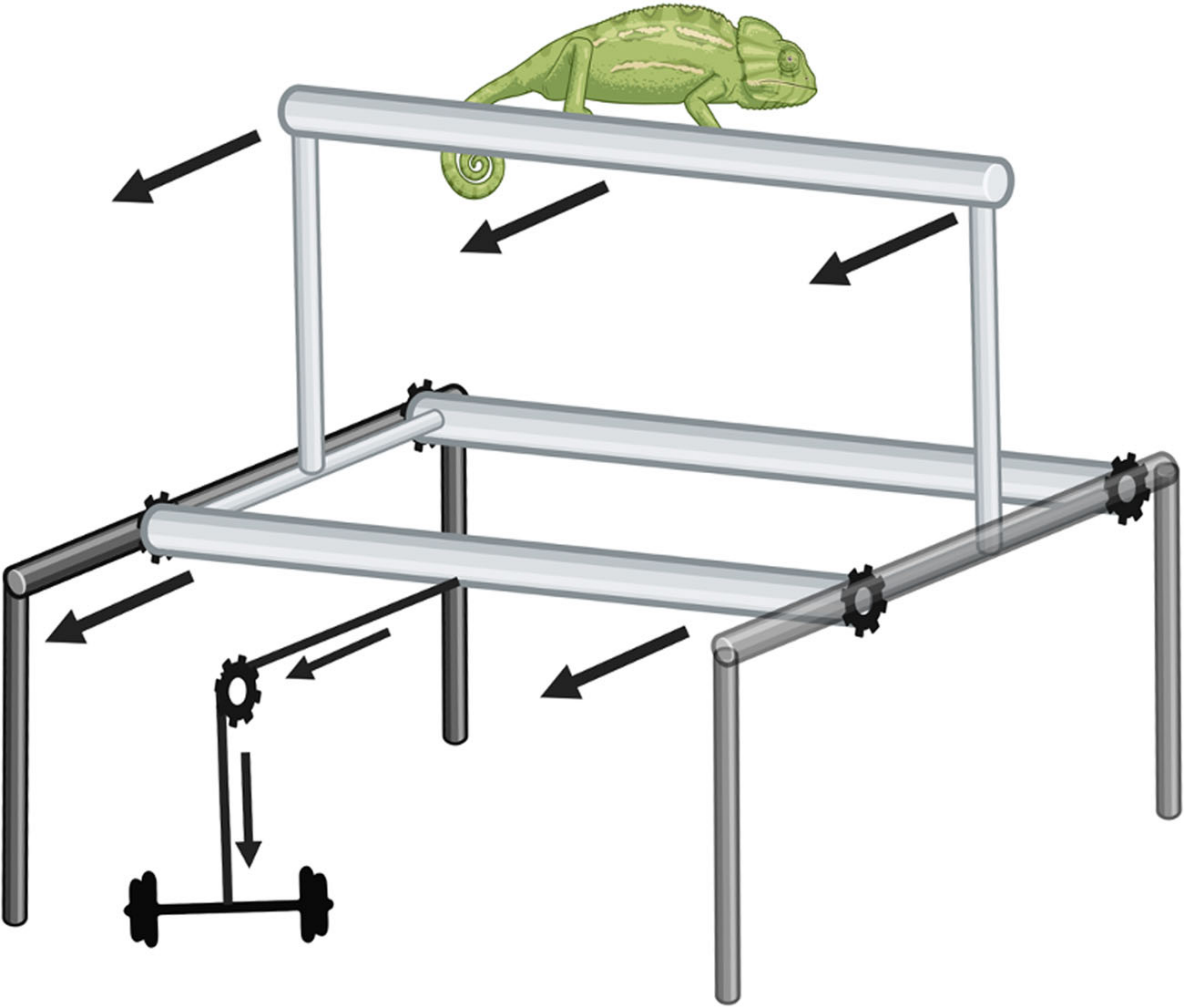
Arboreal perturbation simulation apparatus. Upon release from the holder, the weight (barbell) pulls the perch sled (light gray) laterally via a pulley system. The sled slides relative to the support frame (dark gray) unrestricted until it reaches the end of the tracks on the support frame. Reaching the track end results in a sudden arrest to sled movement, subjecting the animal to a perturbation.

We designed and built a testing apparatus to simulate arboreal perturbations in a repeatable and standardizable manner. This apparatus consisted of a horizontal, beam‐shaped perch (5 cm diameter, 60 cm long) attached on each end by 30 cm vertical posts to a wheeled sled (Figure 2). The wheels of the sled were fitted into horizontal, low friction (aluminum) tracks supported by a wooden frame. The sled could slide horizontally along the track ~23 cm until movement was stopped abruptly at the end of the track. To begin sled movement, a lateral displacement force was applied through an adjustable weighted pulley system. To normalize the forces of the perturbation across our study species, the weight of the applied force was set at six times the average body mass for each species. The perch beam was covered with polyethylene foam to permit more uniform grip and body orientations by all the animals. This foam also prevented the chameleons in particular from completely enclosing the perch within the grip of their zygodactylous digits. Surfaces below the apparatus were highly padded to prevent injury in the event of loss of grip.

### 2.3 Kinematic Data Collection

Nontoxic white paint markings were applied to homologous anatomical landmarks on the appendages, as well as evenly spaced locations along the body axis of each animal. Anatomical landmarks were marked only on the left side, and included wrist, elbow, shoulder, ankle, knee, hip, and five evenly distributed points along the spine, from shoulder to hip. Body center of mass along the length of the body (BCoM) was determined by placing the animal on a beam between two scales (Iijima et al. 2021). The animal was placed on top of the beam and oriented with the anterior end aligned with the end of the beam. We used the equation adapted from Clemente (2014): CoM_SH_ = 100[W_2_ × L/(W_1_ + W_2_) − SSL]/(SHL − SSL), where L is the length of the beam, SHL is the snout–hip length, SSL is the snout–shoulder length, and W_1_ and W_2_ are the masses recorded at the cranial and caudal balances, respectively. A CoM_SH_ of 0 or 100 would indicate the BCoM positioned at the shoulder or hip, respectively. The body axis marking most closely associated with the calculated BCoM location then served as a CoM proxy (“CoM proxy” will be referred to “CoM” throughout the rest of this manuscript). Landmarks were also applied every 2.5 cm for the entire length of the perch. After landmark application, each animal was placed on the apparatus and situated to ensure that the body was on the upward‐facing portion of the perch. Once animals were positioned and all locomotor movement was arrested, the weight attached to the sled was released from its holder and the perch sled would slide toward the end of its track, resulting in a sudden arrest of sled movement and subjecting the animal to a perturbation. This sudden perch sled arrest typically entailed a diminishing lateral oscillation as its inertia settled, which challenged animals to maintain grip in addition to the initial impact. These behaviors were recorded (100 Hz) with two digitally synchronized Phantom v.5.1 high‐speed cameras (Vision Research, Wayne, NJ, USA) oriented at oblique angles to the apparatus. Each animal was subjected to three perturbation trials over the course of a 10‐min recording session before being returned to its original enclosure. Total session and trial numbers varied between individuals due to different levels of subject recalcitrance.

### 2.4 Kinematic Analysis

Videos collected from perturbation trials were calibrated and coordinate data for landmarks were collected using the DLTdv8 application in MATLAB (Hedrick 2008). A standard reduction of the number of frames analyzed was applied to each video to include only relevant frames, from the impact of the perch sled at the end of its slide, plus the immediately following thirty frames (0.3 s). To ensure that the movements analyzed included only movement of the animals, three‐dimensional coordinate data were corrected to remove the minor movements of the perch sled at the end of the perturbation impact event. These normalized data were used to evaluate the movements of the limbs and CoM. Kinematic metrics for the entire trial of body‐height adjusted total CoM displacement (in body heights), elbow angle, and knee angle were calculated from coordinate data using custom code in R Statistical Software (package: rgl, R Core Team 2025).

Kinematics were further analyzed using vector analyses. In these analyses, CoM displacement, elbow angle, and knee angle values derived from each of the 30 frames were used to generate vectors with 30 dimensions. The angle between pairs of these vectors could then be calculated using standard equations (Cullen et al. 2013; Hamilton 1989). An angle near 0° indicates two nearly identical vectors (i.e., two nearly identical kinematic profiles), whereas an angle near 90° indicates perpendicular trajectories (profiles that are not correlated or are independent of each other). These calculations were performed in Microsoft Excel for parallel comparisons (Table 1).

**Table 1:** Multi‐vector kinematic profile divergence comparisons for the numeric kinematic metrics.

Body Configuration	CoM Trajectory Deviation (°)	Elbow Angle Trajectory Deviation (°)	Knee Angle Trajectory Deviation (°)
*Anolis* versus	**18.14**	2.75	1.57
*Chamaeleo*			
*Anolis* versus	4.64	5.64	1.33
*Gastropholis*			
*Chamaeleo* versus	**16.33**	6.55	2.66
*Gastropholis*			

*Note:* Comparisons closer to 0° indicate similar overall kinematic profiles, whereas profile comparisons closer to 90° indicate very different kinematic profiles. Comparisons with the greatest differences (> 15°) are shown in bold.

### 2.5 Tail Behavior Evaluation

Tail behavior during the trials was evaluated by categorization into five different qualitative definitions: “counterbalance”, “dynamic tail rotation”, “loose prehensile wrapping”, “tight prehensile wrapping”, and “no stabilizing behavior”. These categories are based on the diversity of arboreal tail behaviors described in deer mice (Hager and Hoekstra 2021). Specific to this study, “counterbalance” tail behavior denotes behavior where the tail is hung below the perch, presumably drawing the CoM closer (or even below) the perch; “dynamic tail rotation” denotes a swinging of the tail in the transverse plane in reaction to a perturbation, often serving to counteract the inertial force experienced by the proximal portions of the body and, thus, maintaining body position on top of the perch; “loose prehensile wrapping” denotes tail behavior in which the tail is wrapped loosely around the perch, but does not provide any resistance to the perturbation; “tight prehensile wrapping” is identical to the previous category, but providing resistance to the perturbation; “no stabilizing behavior” reflects that no behavior was observed that fell into these categories, nor appeared to contribute to any stabilization after perturbation.

### 2.6 Statistics

Our primary metric of stability was how far the CoM displaced from its pre‐perturbation location. Each CoM displacement was normalized for each animal's body height at the CoM. We constructed linear mixed‐effects models to evaluate how CoM displacement was influenced by five fixed effects and their interactions. These fixed effects are: body configuration (combination of stance and tail prehensility), elbow angle, knee angle, tail behavior category, and restabilization time (defined as the number of frames between CoM maximum displacement and post‐perturbation minimum). Individual animal behavior was assigned as a random effect. All possible sub‐models were ranked using ΔAIC, a metric evaluating the difference between one model's Akaike's information criterion (AIC) and the lowest overall AIC. The best models were then used to calculate the estimated marginal means (package: emmeans, R Core Team 2025).

## 3 Results

**Figure 3:**
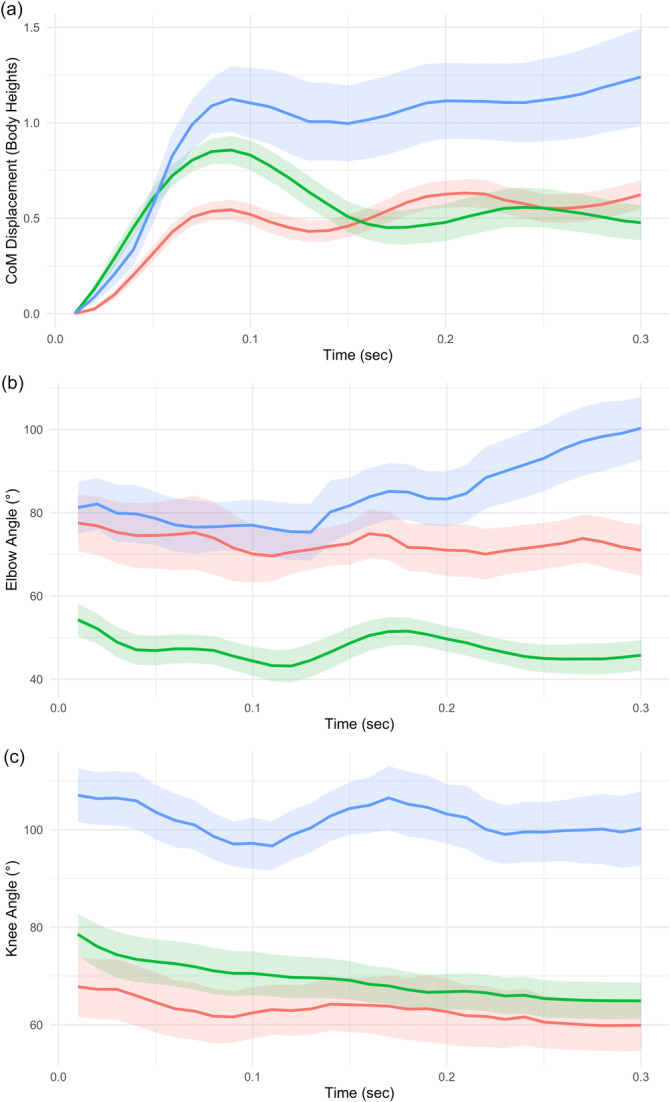
Mean kinematic profiles for (a) CoM displacement, (b) Elbow angle, and (c) Knee angle for all perturbation trials for each of three lizard species. Red represents *Anolis equestris* (sprawling, non‐prehensile); blue represents *Gastropholis prasina* (sprawling, prehensile), and green represents *Chamaeleo calyptratus* (upright, prehensile). Dark lines represent means, shading represents SEM. *N* = 3 individuals per each species, with 4–11 trials per species. Data represented here has occurred immediately after a perturbation.

Videos showing example trials of our representative species are available in the Supporting Material. Our first set of comparisons assessed the species averages for CoM displacement and elbow and knee kinematics over the thirty frames immediately after the perturbation (Figure 3). *Gastropholis* had the greatest average height‐adjusted CoM displacement (Figure 3A) and was significantly different from that of both *Anolis* and the *Chamaeleo* (*p* < 0.001). CoM displacement did not differ significantly between *Anolis* and *Chamaeleo*. For elbow angle (Figure 3B), *Chamaeleo* was significantly lower in magnitude than both the species with sprawling configurations (*p* < 0.001). There were no significant differences between elbow angles in the sprawling configurations. With knee angle (Figure 3C), all three body configurations' averages were significantly different from one another (*p* < 0.001), with *Gastropholis* showing the highest values and *Anolis* the lowest.

Our second set of comparisons evaluated the similarity of overall kinematic profiles. For CoM displacement, each lizard first tilted toward the direction of sled travel due to inertia, then returned towards its original position as a stabilizing response. Overall kinematic profiles were most similar between the two species using sprawling configurations, with a divergence angle of only 4.6° between the kinematic profile vectors (Table 1). CoM kinematic profile divergence was much greater between upright *Chamaeleo* and each of the other two species (16–18° across both comparisons, Table 1). Comparisons of elbow and knee kinematics were more similar across all three species, with kinematic divergence angles between only 1–7° (Table 1).

**Figure 4:**
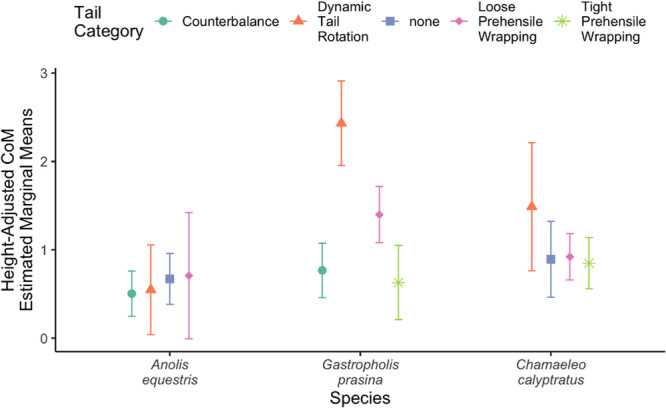
Estimated marginal means of CoM Displacement using factors in the model with the lowest AIC (body configuration + tail behavior).

The best linear mixed‐effects model to explain CoM Displacement is the model with body configuration and tail behavior as the fixed effects, and the individual animal as a random effect (CoM Displacement ~ Body Configuration * Tail Behavior + (1|Animal)). Other models had ΔAIC values greater than 2, suggesting (Burnham et al. 2011) that this best model is far superior in its fit of the data than other sets of variables (Table 2). Dynamic tail rotation tail behavior in *Gastropholis* was found to have a significantly greater estimate of CoM displacement, whereas the other tail behaviors across the body configurations did not yield any significant differences (Figure 4).

**Table 2:** Linear mixed‐models evaluated using AIC.

# of Factors	Model	AIC	ΔAIC	R2m	R2c
Fully factorial	CoM Displacement~Body Configuration*Elbow Angle*Knee Angle*Tail Behavior*Restabilization Time + (1|Animal)	464.38	385.28	0.73	0.73
4 Factor model	CoM Displacement~Body Configuration*Elbow Angle*Knee Angle*Tail Behavior + (1|Animal)	323.26	244.17	0.75	0.76
	CoM Displacement~Body Configuration*Elbow Angle*Knee Angle*Restabilization Time + (1|Animal)	328.78	249.69	0.34	0.34
	CoM Displacement~Body Configuration*Elbow Angle*Tail Behavior*Restabilization Time + (1|Animal)	245.49	166.39	0.68	0.68
	CoM Displacement~Body Configuration*Knee Angle*Tail Behavior*Restabilization Time + (1|Animal)	243.08	163.98	0.66	0.66
	CoM Displacement~Elbow Angle*Knee Angle*Tail Behavior*Restabilization Time + (1|Animal)	421.86	342.76	0.67	0.71
3 Factor model	CoM Displacement~Body Configuration*Elbow Angle*Knee Angle + (1|Animal)	200.97	121.88	0.34	0.34
	CoM Displacement~Body Configuration*Elbow Angle*Tail Behavior + (1|Animal)	162.58	83.49	0.70	0.70
	CoM Displacement~Body Configuration*Elbow Angle*Restabilization Time + (1|Animal)	172.97	93.88	0.32	0.32
	CoM Displacement~Body Configuration*Knee Angle*Tail Behavior + (1|Animal)	159.80	80.71	0.68	0.68
	CoM Displacement~Body Configuration*Knee Angle*Restabilization Time + (1|Animal)	173.15	94.05	0.28	0.33
	CoM Displacement~Body Configuration*Tail Behavior*Restabilization Time + (1|Animal)	114.84	35.74	0.62	0.62
	CoM Displacement~Elbow Angle*Knee Angle*Tail Behavior + (1|Animal)	240.80	161.70	0.67	0.67
	CoM Displacement~Elbow Angle*Knee Angle*Restabilization Time + (1|Animal)	187.94	108.85	0.14	0.22
	CoM Displacement~Elbow Angle*Tail Behavior*Restabilization Time + (1|Animal)	225.57	146.48	0.28	0.41
	CoM Displacement~Knee Angle*Tail Behavior*Restabilization Time + (1|Animal)	213.48	134.39	0.46	0.47
2 Factor model	CoM Displacement~Body Configuration*Elbow Angle + (1|Animal)	123.29	44.20	0.31	0.31
	CoM Displacement~Body Configuration*Knee Angle + (1|Animal)	126.71	47.61	0.25	0.30
	CoM Displacement~Body Configuration*Tail Behavior + **(1|Animal)**	**79.09**	**0.00**	**0.61**	**0.61**
	CoM Displacement~Body Configuration*Restabilization Time + (1|Animal)	111.12	32.03	0.26	0.38
	CoM Displacement~Elbow Angle*Knee Angle + (1|Animal)	136.15	57.06	0.11	0.23
	CoM Displacement~Elbow Angle*Tail Behavior + (1|Animal)	144.37	65.27	0.29	0.38
	CoM Displacement~Elbow Angle*Restabilization Time + (1|Animal)	132.16	53.06	0.02	0.22
	CoM Displacement~Knee Angle*Tail Behavior + (1|Animal)	135.89	56.79	0.44	0.44
	CoM Displacement~Knee Angle*Restabilization Time + (1|Animal)	125.49	46.40	0.13	0.23
	CoM Displacement~Tail Behavior*Restabilization Time + (1|Animal)	120.97	41.88	0.27	0.38
1 Factor model	CoM Displacement~Body Configuration + (1|Animal)	96.76	17.67	0.23	0.31
	CoM Displacement~Elbow Angle + (1|Animal)	112.20	33.10	0.01	0.18
	CoM Displacement~Knee Angle + (1|Animal)	106.18	27.09	0.10	0.24
	CoM Displacement~Tail Behavior + (1|Animal)	94.43	15.34	0.28	0.36
	CoM Displacement~Restabilization Time + (1|Animal)	107.10	28.01	0.01	0.22

*Note:* Best model to explain CoM Displacement is that with body configuration and tail behavior as the fixed effects (bolded).

## 4 Discussion

Comparisons across our three lizard species, representing three different arboreal body configurations, shed light on how morphological and behavioral features contribute to resisting environmental perturbations in arboreal habitats. When considering only mean height‐adjusted CoM displacement, *Gastropholis* showed significantly greater displacements than species using the other two configurations we evaluated. Comparisons of the kinematic profiles of these CoM displacements (Figure 3, Table 1) indicated that the mean height‐adjusted CoM displacement of the *Chamaeleo* had the most distinct kinematic profile of CoM movement, appearing to show a consistent restabilization back toward the original CoM position. However, comparisons of limb joint angles through the trials showed a different pattern. Kinematic profile vectors for these metrics exhibited minimal differences across the three species (Table 1), suggesting that these joint angles show similar patterns of change during perturbations across the body configurations we compared. However, average elbow angles throughout the trial were significantly lower for the *Chamaeleo* than for other species, and average knee angles were greatest for the *Gastropholis* (Figure 3B,C). These patterns indicate distinct conformations between the forelimb and hindlimb across our focal body configurations, potentially contributing to distinct stabilization behaviors across these species.

Comprehensive linear mixed‐models indicated that body configuration and tail behavior (as well as individuals as a random effect) were the factors that best explained variation in CoM displacement across our trials. This indicates that elbow angle, knee angle, and restabilization time may not have strong effects on CoM displacement. When considering the sub‐model that provided the strongest explanation of our data, the effect of tail behavior showed an interesting pattern: dynamically rotating the tail was associated with greater average CoM displacements for the sprawling, prehensile‐tailed *Gastropholis*. (Figure 4). Although these patterns are associated, it is difficult to resolve whether dynamic tail rotation is a stabilizing behavior that tends to be executed in instances of great instability, or whether dynamic tail rotation is a “last‐ditch” stabilization behavior that is chaotic and unstable in and of itself, eliciting greater CoM displacement in the process. For other tail behaviors, CoM displacement was essentially consistent regardless of the stabilizing behavior implemented, there is greater variation in CoM across tail wrapping behaviors in *Gastropholis* (Figure 4). Beyond high displacement values associated with dynamic tail rotation, *Gastropholis* showed that trials with tight prehensile wrapping typically had less CoM displacement than trials with loose prehensile wrapping, indicating that the grip of the tail on the perch does impact stability for this specific body configuration. The variation in tail behaviors employed across specific ranges of CoM displacements in *Gastropholis* might signify that certain tail behaviors are employed at different levels of instability in this species.

Results from our comparisons show that arboreal animals may use a range of different kinematic strategies to restabilize, though these do not limit CoM displacement equally well. These differences may reflect differences in foraging and navigation of habitat across the species we compared. *Gastropholis* (with the sprawling, prehensile‐tailed body configuration), when not sequestered in tree hollows, are very active animals, and observations from our captive animals show climbing and crawling with little pause as they explore their habitat. Cognitive evaluations have shown a strong capacity for spatial learning in lacertids (De Meester et al. 2022). *Anolis equestris* are regarded as a crown‐giant ecomorph of the *Anolis* genus, often situated in tree canopies and patrolling their territories (Williams 1983; Nicholson and Richards 2011). These foraging and habitat navigation methods differ from those of *Chamaeleo*. The three lizard species we compared may accommodate instability in different ways: chameleons and anoles may preferentially maintain a firm grip on the substrate, whereas *Gastropholis* lizards may be less specialized for gripping of substrates and, instead, manage displacement via dynamic strategies. These results illustrate that across the diverse morphologies and behaviors evident in arboreal animals, factors such as redundancy and many‐to‐one mapping (Wainwright 2005) may enable multiple strategies to achieve similar performance capabilities associated with stabilization. In conclusion, body configuration and tail behavior are predictive of some level of ability to contend with perturbations, though these metrics may be compensated for via other behaviors associated with foraging habits.

## Conflicts of Interest

The authors declare no conflicts of interest.

## Supporting information

anolis_perturbation.

chamaeleo_perturbation.

gastropholis_perturbation.

## Data Availability

The data that support the findings of this study are openly available in figshare at https://doi.org/10.6084/m9.figshare.30121252.v1.
